# Cytotoxic lymphocytes-related gene ITK from a systematic CRISPR screen could predict prognosis of ovarian cancer patients with distant metastasis

**DOI:** 10.1186/s12967-021-03119-3

**Published:** 2021-10-26

**Authors:** Mengyao Xu, Shan Huang, Jiahui Chen, Wanxue Xu, Rong Xiang, Yongjun Piao, Shuangtao Zhao

**Affiliations:** 1 School of Medicine, Nankai University, Tianjin, 300071 China; 2 Tianjin Key Laboratory of Human Development and Reproductive Regulation, Nankai University Affiliated Hospital of Obstetrics and Gynecology, Tianjin, China; 3 Department of Nuclear Medicine and PET/CT-MRI Center, The First Affiliated Hospital of Jinan University, Tianhe District, 613 West Huangpu Road, Guangzhou, 510630 China; 4 Department of Thoracic Surgery, Beijing Tuberculosis and Thoracic Tumor Research Institute/Beijing Chest Hospital, Capital Medical University, Beijing, 101149 China

**Keywords:** Ovarian cancer, Metastasis, CRISPR screen, Bioinformatic analysis

## Abstract

**Background:**

Ovarian cancer, a highly metastatic malignancy, has benefited tremendously from advances in modern human genomics. However, the genomic variations related to the metastasis remains unclear.

**Methods:**

We filtered various significant genes (n = 6722) associated with metastasis within a large-scale functional genomic CRISPR/Cas9 knock-out library including 122,756 single guide RNAs, and identified *ITK* (IL2 Inducible T Cell Kinase) as a potential cancer suppressor gene for ovarian cancer metastasis. Downstream bioinformatic analysis was performed for ITK using public databases.

**Results:**

We found that patients in low-ITK group had poor prognosis and more distant metastasis than those in high-ITK group in TCGA and GEO databases. We also demonstrated that *ITK* combined with the clinical factors could accurately predict prognosis through multiple Cox regression analysis and ROC analysis. Moreover, alterations correlated with distant metastasis emereged with significantly increased expression in *SAMRCD1* in low-ITK group, but *CD244* and *SOCS1* in high-ITK group. Integrated analysis revealed dysregulated molecular processes including predominantly oncogenic signaling pathways in low-ITK group but immune related pathways in high*-*ITK group, which suggested ITK might inhibit distant metastasis in ovarian cancer. Furtherly, deconvolution of the cellular composition of all samples validated the close correlation between ITK and immune related function especially for cytotoxic lymphocytes.

**Conclusions:**

Together, these data provide insights into the potential role of ITK, with implications for the future development of tansformative ovarian cancer therapeutics.

**Supplementary Information:**

The online version contains supplementary material available at 10.1186/s12967-021-03119-3.

## Introduction

Ovarian cancer, a heterogeneous neoplasm, could not be screened early and 80% of them were typically diagnosed in the late stage. Globally, a total of 21,750 new cases are diagnosed as ovarian cancer each year, with 13,940 cancer-specific deaths [1–3]. Notably, peritoneal spread was reported as the primary metastasis in ovarian cancer [4], in which malignant cells circulated in the peritoneal fluid to produce the later spread in abdominal organs [5]. The previous study reported that the cancer stem cell-like properties play a positive role in metastatic spread [6]. In the past decades, various treatments were applied to improve the high mortality but failed, such as the oral contraceptives and hormone replacement therapy [7, 8], genetic molecular alterations [9, 10], immunotherapy, chemotherapy, and the inhibitor based treatment including Platinum-based chemotherapy and poly (ADP-ribose) polymerase inhibitors [11]. Therefore, a new treatment was urgently to develop to inhibit the spread of cancer cells and then reduce the ovarian cancer-related mortality. Furthermore, the cross-talk between intracellular macrophages and disseminated cancer cells are identified as a new target to reduce metastasis and disease recurrence [12].

Previous studies showed that Interleukin-2-inducible T-cell kinase (ITK) is a member of the TEC family of non-receptor tyrosine kinase, which acts an essential mediator of intracellular signal transduction in both T-cells and natural killer (NK) cells [13–16]. ITK functions as a downstream signaling between T-cell and NK cell surface receptor and regulates multiple aspect of T-cell development and function [17, 18]. It has been demonstrated that the deficiency of ITK is associated with the malfunction of T cell development, and T cell disorderes related human diseases [19]. The numerous evidences of ITK deficiency related human disease have suggested ITK as a promising therapeutic target for various human disease with ITK based molecule inhibitors such as the aminothiazole based ITK inhibitors [20, 21] for suppression of lung inflammation, benzimidazole based ITK inhibitors [22], aminopyrimidine based ITK inhibitor [23], 3-aminopyride-2-ones based ITK inhibitors [24]. In addition to the involvement in inflammatory response and autoimmune diseases, ITK was also involved in oncogenesis[25]. Mutations in *ITK* directly cause defects in T-cell signaling pathways [26]. A loss of function mutation of *ITK* led to the occurrence of Hodgkin and non-Hodgkin lymphoma, mononucleosis, lymphoproliferative disease after infections [27, 28]. Generally, ITK plays an important role in the inflammatory processes [23] and oncogenesis [25], which pave the promising way to develop inhibitors of the ovarian cancer.

This study was to investigate the predicitive role of *ITK* in the prognosis of patients with ovarian cancer. We collected 6,722 significant genes correlated with metastasis within CRISPR/Cas9 library and identified *ITK* as a key factor to predict clinical outcomes of patients with ovarian cancer in the Cancer Genome Atlas (TCGA) and Gene Expression Omnibus (GEO) datasets. Integraged analysis showed that dysregulated molecular processes including predominantly oncogenic signaling pathways are enriched in low-ITK group but immune related pathways in high*-*ITK group. Furtherly, we discovered that *ITK* was positively correlated with *CD244* and *SOCS1* (a suppressor for cancer) but negatively correlated with *SMARCD1*. Additionally, deconvolution of the cellular composition of all samples with MCP-counter and CIBERSORT methods validated the immune related analysis and discovered the close correlation between *ITK* and cytotoxic lymphocytes. Our results provided a new method for ovarian cancer diagnosis and treatement in the clinical practice.

## Methods

### Patients and samples

All the clinical and genomic data was collected from TCGA and GEO databases. A total of 887 ovarian cancer samples and 12 normal controls were selected into this study, including 379 patients samples from TCGA, 18 patients and 12 normal samples from GSE38666, 380 patients samples from GSE140082, 110 patients samples from GSE17260, respectively. These samples enrolled into this study were collected between March 2010 and November 2019 (Additional file 1: Table S1).

### CRISPR/Cas9 knockout in vivo screening and mouse model development

SKOV3 cells were purchased from the Cell Bank of the Chinese Academy of Sciences (Shanghai, China). SKOV3 was cultured in McCoy’s 5A Medium Modified (Biological Industries, Israel). Genome-scale CRISPR knock-out (GeCKO) v2.0 pooled library was purchased from Addgene (Watertown, MA, USA) and amplified as described [29]. The library totally was composed of 122,756 sgRNAs, including 1,000 control sgRNAs, targeting 19,050 genes and 1,864 miRNAs. SKOV3 cells were generated and infected with the library carried by Lentivirus, and the multiplicity of infection (MOI) was around 0.5. Then SKOV3 cells expressing sgRNAs were selected with puromycin, 1 × 10^6^ cells were orthotopically injected into NOD-SCID mice. Tumor growth were monitored in every 3–4 days and mice sacrificed after 40 days when tumor burden was evident or general health conditions were failing. Primary tumors and metastatic nodules from different organs in the peritoneal cavity were dissected and isolated in vitro expansion culture for the second round of orthotopic injection, respectively. After 3 rounds selection in an in vivo metastasis model, primary cells and metastatic cells were collected for subsequent high throughput DNA deep sequencing to identify metastasis-related candidate genes. We used the RNAi Gene Enrichment Ranking (RIGER) *P*-value to analyze sgRNAs that were significantly enriched in the metastatic group (sgRNA_Met_) or orthotopic group (sgRNA_Pri_) in ovarian cancer mouse model. The screening criteria were the number of enriched sgRNA targeting each gene ≥ 2, P-value < 0.05 and the normalized enrichment score (NES) < − 1.2.

Female NOD-SCID mice with 6–8 weeks old were purchased from SPF Biotechnology (Beijing, China). Human SKOV3 ovarian cancer cells 1 × 10^6^ cells in 10μL sterile PBS orthotopically injected into left ovary of NOD-SCID mice. Ascites volumes, the numbers of metastatic nodules were measured and total cells in ascites were harvested at 40 days post-inoculation for xenograft model. Metastatic behavior was also determined by Caliper Life Science IVIS Lumina II Spectrum imaging system. After 3 rounds of in vivo screening, the behavior of cells were monitored at Day 8, Day 14 and Day 21 from the time of tumor implantation.

### Tumor mutation and biological function analysis

Mutation data was downloaded from cBioPortal for Cancer Genomics (https://www.cbioportal.org/), and then submitted the query regarding “ITK” in the input box of MutationMapper. The lollipop plot was generated according to the unique paitent ID.

To retrieve the biological function of *ITK*, the GSEA anlaysis (https://www.gsea-msigdb.org/gsea/index.jsp) was performed in ovarian cancer by using TCGA dataset. Interested genes (n = 4,469) were adopted to process by GSEA against the curated gene sets (Hallmark, KEGG and GO) from the molecular signature database (MSigDB) to investigate the significant enrichment of signaling pathways (FDR < 0.05 and minimum gene set size ≥ 3). Then, we used R package *tidyverse* to visualize these results. Meanwile, we used Metascape tool (https://metascape.org/gp/index.html#/main/step1) to complete the functional network of these significant genes.

We applied two deconvolution methods—CIBERSORT algorithm [30] to estimate the relative cellular fraction of 22 immune cell types, and MCP-counter [31] software to compute the absolute abundance scores of 8 major immune cell types (CD8^+^ T cells, CD3^+^ T cells, Natural killer (NK) cells, cytotoxic lymphocytes, B lymphocytes, myeloid dendritic cells, monocytic lineage cells and neutrophils), fibroblasts and endothelial cells. We input log2-transformed genes expression value for these two methods, and used the LM22 leukocyte gene signature as the input gene signature for CIBERSORT. The deconvolution profiles were then clustered hierarchically and compared between low and high-ITK groups.

### Statistical analysis

The raw RNA data was downloaded from TCGA data set and then processed by *limma* package to identify DEGs (|Fold Change|≥ 2 and FDR *q*-value < 0.05) between regional and distant metastasis subgroup. We defined a mean as the cutoff value in the expression of *ITK* in each set. We applied Pearson’s chi-square to identify the statistical significance in the basic characteristics. The *t* test was used to distinguish the distributive difference of *ITK* expression between low and high groups. The correlation analysis between *ITK* and the other 4,469 genes was evaluated with Pearson correlation analysis in the TCGA cohort. We applied ROC curves (R package: *timeROC*) analysis to identify the sensitivity and specificity of *ITK* and other clinical factors. We quantified the performance of these factors with the area under ROC curve. All the factors were evaluated based on their values from the TCGA and GEO data sets, separately. In the overall survival (OS) analysis, the Kaplan–Meier method was performed to explore the relationship between risk factors and overall survival, and compare the survival curves with log-rank test. Then we used multiple Cox proportional hazards regression analysis to evaluate the independency of this gene. Hazard rations (HRs) and 95% confidence intervals (CIs) were produced in each cohort. We used R software (version 4.0.2) to perform all the statistical analysis, and defined *p* < 0.05 as the significant threshold.

## Results

### Screen the significant genes associated with metastasis by using CRISPR/Cas9 library

**Fig. 1:**
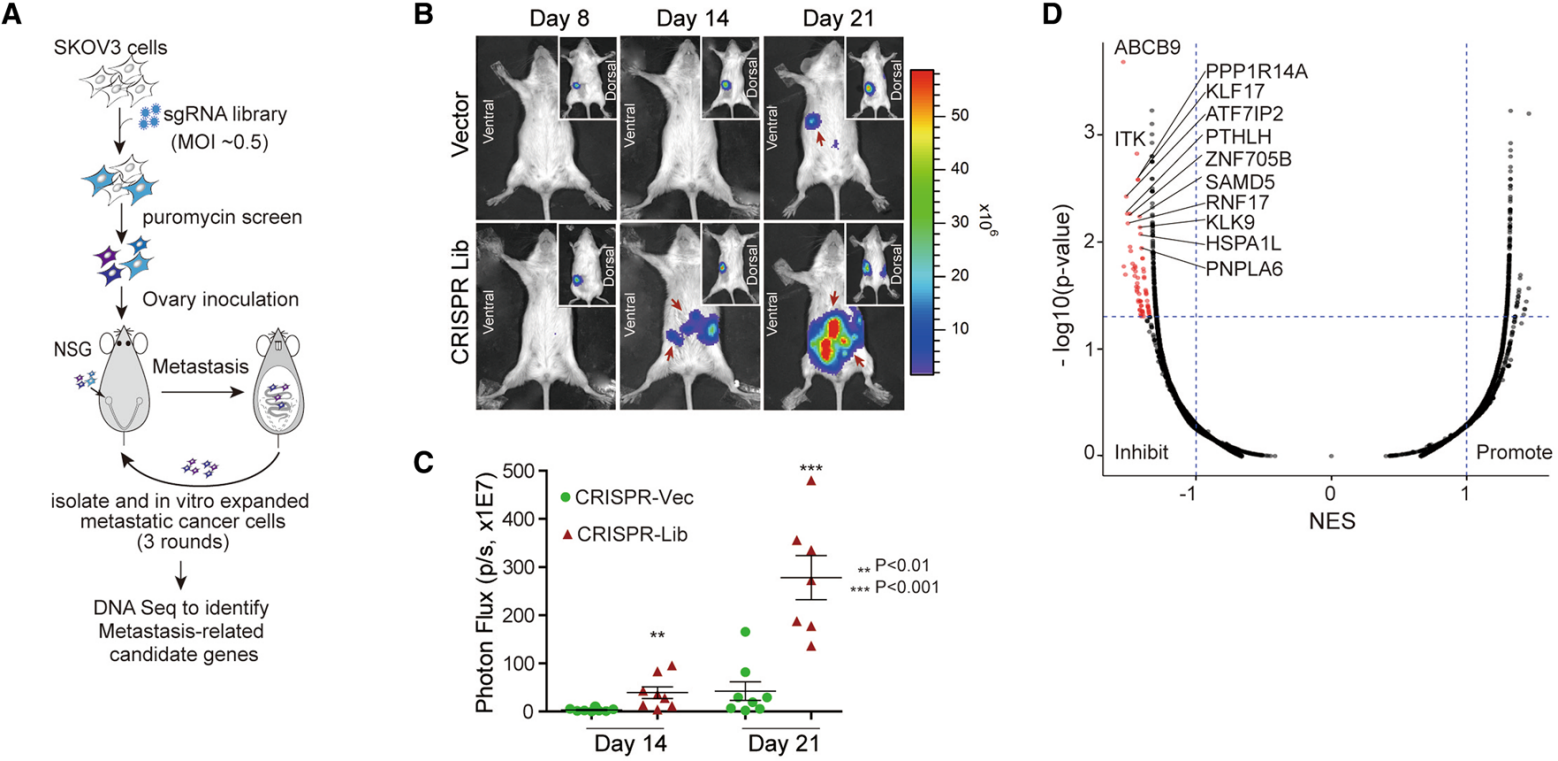
Establishment of orthotopic transplant tumor model in NOD-SCID mice and high-throughput screening of genes associated with metastasis using CRISPR/Cas9 library, **A** Flow chart of ovarian cancer orthotopic mouse model construction. **B** In vivo imaging of CRISPR-Lib and CRISPR-Vec at different stage, n = 18. **C** Photo Flux change of CRISPR-Lib and CRISPR-Vec,** p < 0.01, *** p < 0.001. **D** Volcano plot of metastasis-related candidate genes. Red dots represent inhibit genes in ovarian tumor metastasis model

To investigate the significant genes correlated with metstasis, we established the orthotopic transplant tumor model in NOD-SCID mice with relatively low metastatic capacity human EOC cell SKOV3. We first transfected the human CRISPR knockout library (GeCKO v2.0) into SKOV3 cells, the CRISPR knockout library contains 19,050 sgRNAs that specifically targeting protein encoding genes, along with 1864 miRNAs and 1000 non-targeted sgRNAs [32]. Subsequently, we collected orthotopic tumor tissues and metastatic tissues from the surface of various organs including ascites in the peritoneal cavity, isolated and expanded the culture in vitro for the next round of orthotopic transplant (Fig. 1A). In vivo imaging showed the growth and metastasis of tumors in CRISPR Vec and CRISPR Lib on days 8, 14 and 21 at the third round, it was observed that on days 14 and 21, NOD-SCID mice in CRISPR Lib developed significant metastasis on the ventral side, whereas intraperitoneal tumors were confined in orthotopic and did not metastasize (Fig. 1B). Furthermore, we observed the significant changes of Photon Flux in CRISPR Lib and CRISPR Vec at day 14 (*p* < 0.01) and day 21 (*p* < 0.001; Fig. 1C), which indicated that SKOV3 cells transduced with the human CRISPR knockout library had strong ability of tumor metastasis compared with CRISPR Vec. The numbers of metastatic nodules on the surfaces of intestines were shown in Additional file 2: Figure S1A. Bioluminescence indicated the presence of tumors from the primary site in CRISPR Vec and presence of tumors from the primary site and intestines in CRISPR Lib (Additional file 2: Figure S1B). After 3 rounds of in vivo screening, highly metastatic SKOV3 cells were performed to high-throughput sgRNA ligbrary sequencing. The RIGER P analysis was used to analyze the data of sgRNA library sequencing, As a result, a total of 91 genes were identified as inhibited or promoted factors for the ovarian tumor metastasis in the established model (Fig. 1D). Notebably, *ITK* was one of the the top three genes to inhibit metastasis in the mouse model of ovarianS cancer. These results demonstrated that *ITK* might be an important tumor supressor gene for ovarian cancer metastasis.

### *ITK* expression was significantly correlated with distant metastasis and prognosis of patients with ovarian cancer

**Fig. 2:**
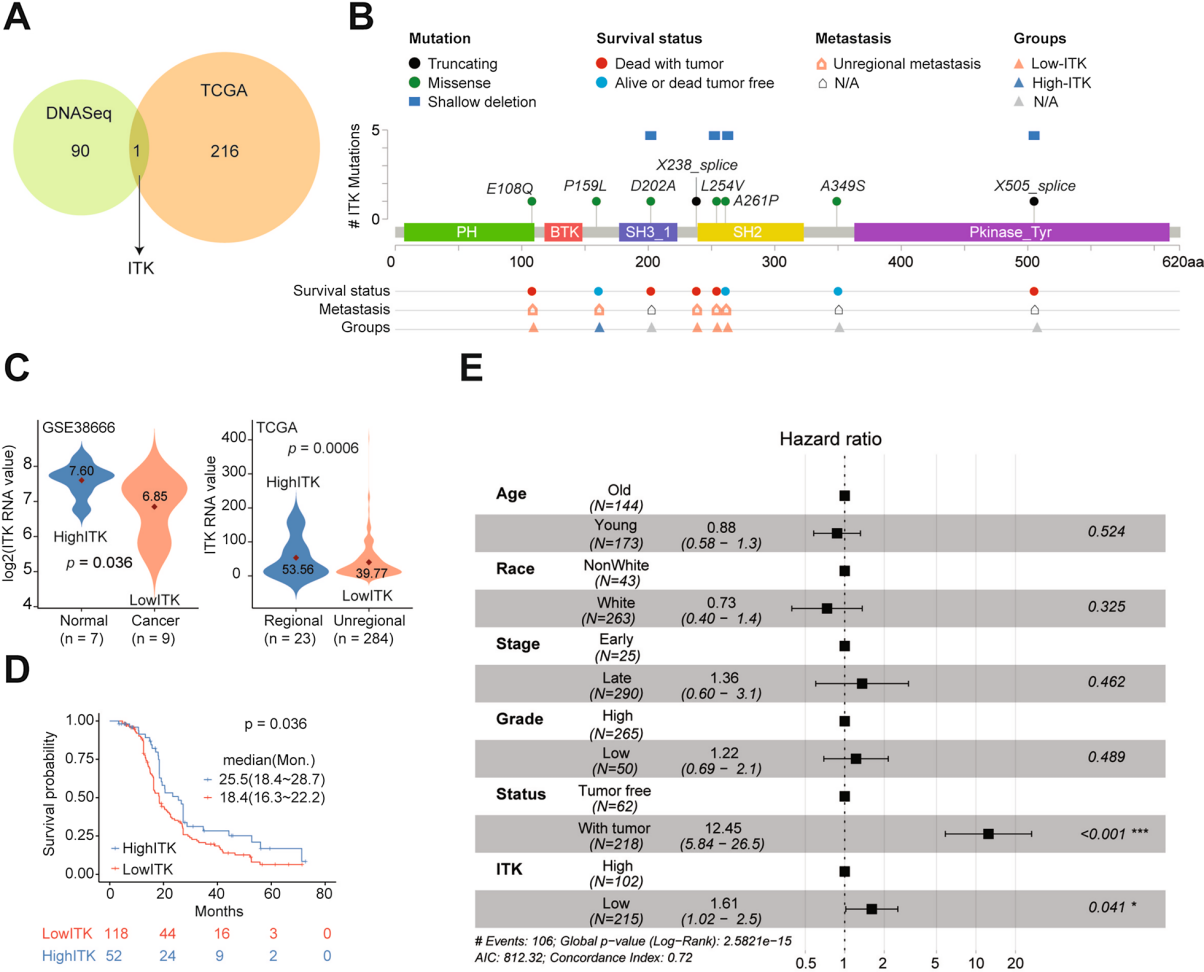
ITK expression was significantly correlated with distant metastasis and prognosis of patients with ovarian cancer. **A** The correlation between DNA sequences and TCGA data set. **B** ITK mutation analysis in ovarian cancer patients. **C** The ITK expression level analysis in GSE38666 and TCGA data sets. **D** Kaplan–Meier Survival Analysis of ITK expression level in TCGA data set, p = 0.036. **E** The hazard ratio evaluation with different clinical factor. (Cox regression model, *p < 0.05, ***p < 0.001)

To explore the correlation between the distant metastasis and the prognosis of ovarian cancer patients, we compared 91 significant genes from DNA-seq filtration with 217 tumor specific genes with significance between regional and distant metastaisis subgroup from TCGA cohort and obtained one overlapped gene *ITK* (Fig. 2A and Additional file 3: Table S2). And then we mapped mutations on a linear protein and its domanis, and found 2 truncating mutations (X238 splice and X505 splice) and 6 missense mutations, of which 4 shallow deletion were produced following with these mutations (Fig. 2B). Meanwhile, we discovered patients with these serious DNA mutation were almost low-*ITK* expression, un-regional metastasis and dead with tumor (Fig. 2B), which could partially explain the low levels of *ITK* expression in tumor tissues and indicate its prognostic role. Then we discovered that *ITK* was with significantly lower expression (*p* = 0.036) in tumor (n = 9) than that in normal (n = 7) samples. Similar result was presented between regional (n = 23) and distant (n = 284) metastasis groups (*p* = 0.0006; Fig. 2C). This result suggested that ITK might inhibit distant metastasis of ovarian cancer. However, these discoveries should be validated by biological experiments.

To further explore the prognostic role of *ITK* in ovarial cancer, we divided these samples into two groups (low and high-ITK group) based on the mean value as a cut off threshold. Then we discovered that patients with low value expression of *ITK* (median survial months: 25.5 (95%CI: 18.4–28.7)) had higher risk than those with high values (median survial months: 18.4 (95%CI: 16.3–22.2); *p* = 0.036; Fig. 2D). Also, multivariate analysis with Cox regression including another 5 prognostic factors revealed that *ITK* expression was significantly associated with OS as a continuous variable in TCGA cohort (HRs = 1.61 (95%CI: 1.02–2.50); *p* = 0.041; Fig. 2E). These results confirmed the ability of *ITK* in predicting survival as an independent factor.

### Valiation of *ITK* for survival prediction in GEO data sets

**Fig. 3:**
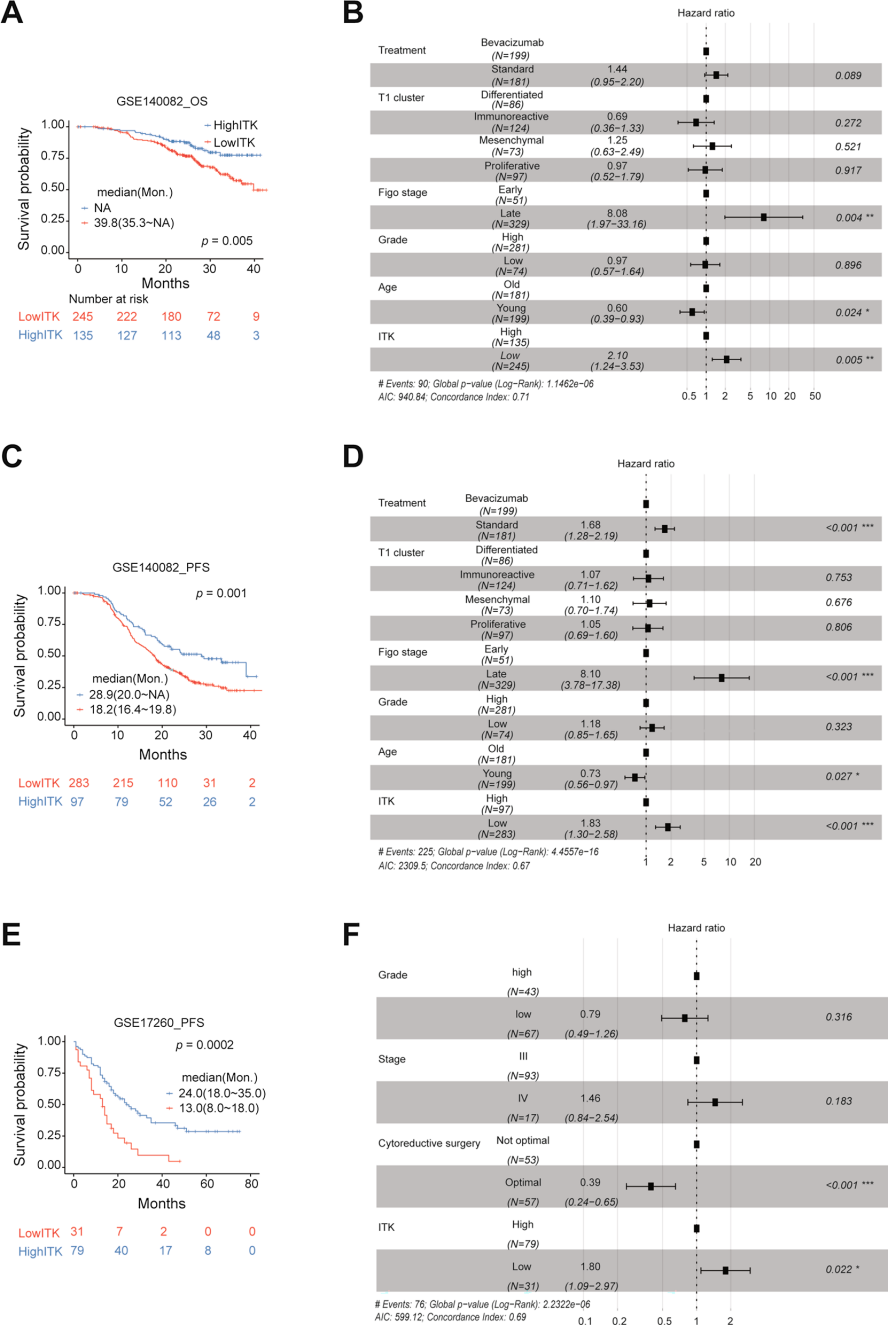
Validation of ITK for survival prediction in GEO data sets. Kaplan–Meier Survival Analysis of ITK expression level in GSE140082 (**A**), GSE140082 (**C**), GSE17260 (**E**). The hazard ratio evaluation with different clinical factor (**B**, **D** and **F**, Cox regression model, *p < 0.05, **P < 0.01, ***p < 0.001)

To validate our discoveries, we selected another two GEO data sets (GSE140082 and GSE17260) to evaluate the prognostic power of *ITK*. Similarly, we classified patients of each cohort into two subgroups (low and high value) based on the mean value as the cutoff point. In GSE140082, patients with low expression values had significantly poor clinical outcomes (median survial months: NA for OS and 28.9 (95%CI:20.0-NA) for PFS) than those with high expression values (median survial months: 39.8 (95%CI: 35.3-NA) for OS and 18.2 (95%CI:16.4–19.8) for PFS; *p* < 0.05; Fig. 3A, C). Then, multivariate analysis with Cox regression including another 5 prognostic factors reveled that *ITK* expression was significantly associated with OS or PFS as a continuous variable in this cohort (HRs > 1.80; *p* < 0.05; Fig. 3B, D). Finally, we generated the similar results as above in GSE17260 (Fig. 3E, F). Integrately, *ITK* could predict prognosis of patients with ovarian cancer independently as a favorable biomarker.

### *ITK* had strong diagnositic power in the prognostic prediction

**Fig. 4:**
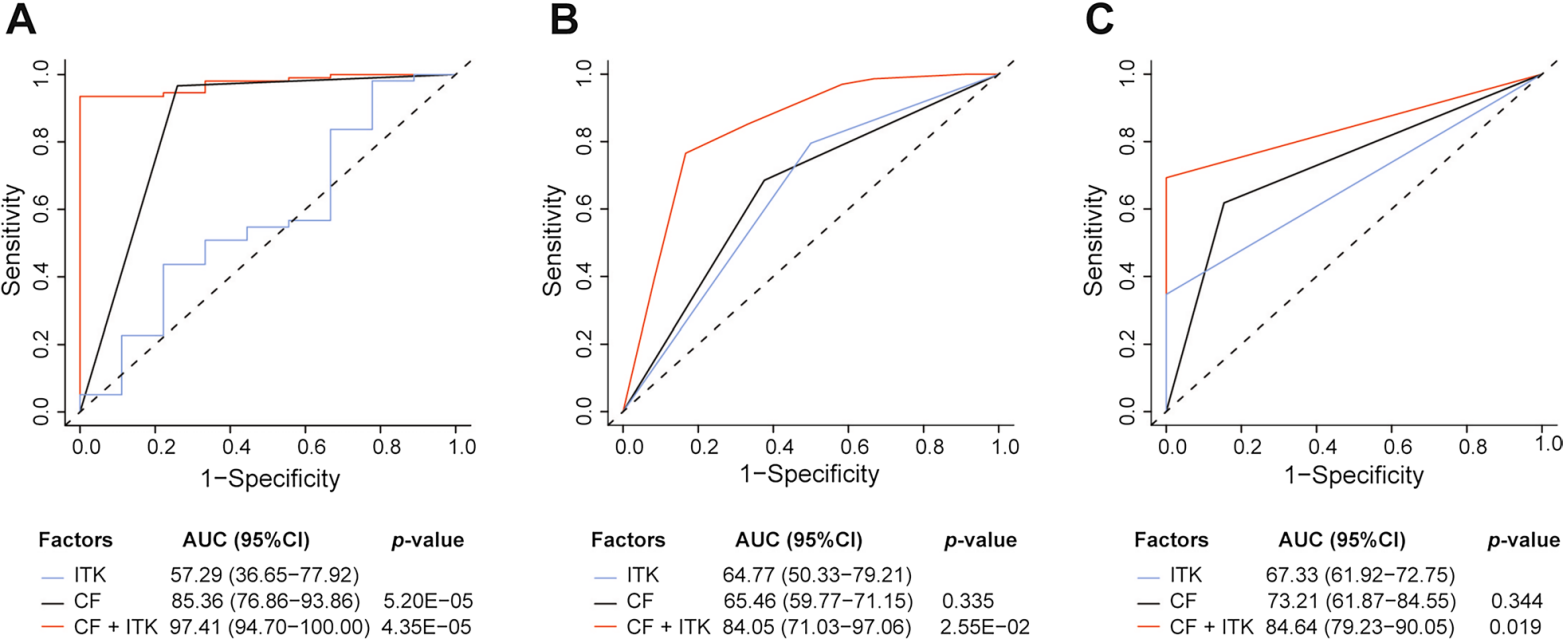
ROC curves compare the prognostic power of ITK with clinical risk factors in TCGA and GEO data sets. **A **TCGA. **B** GSE140082. **C** GSE17260. P-values show the AUC value of ITK vs. the ones of another features. *ROC* receiver operator characteristics, *AUC* area under curves, *CI* confidence interval

To confirm its predictive power in prognosis correlated with metastasis, we performed ROC analysis and computed the area under curve (AUC) value for the significant clinical factors and *ITK* expression. Although the AUC value of *ITK* was lower than the clinical factors (CF), *ITK* combined CF could reach significantly higher AUC values than any single AUC value from CF (AUC > 0.84; *p* < 0.05; Fig. 4A–C). These data indicated that ITK could add diagnostic power to the clinicophological prognostic features.

### Dysregulated signaling pathways of *ITK* between two subgroups with different prognosis

**Fig. 5:**
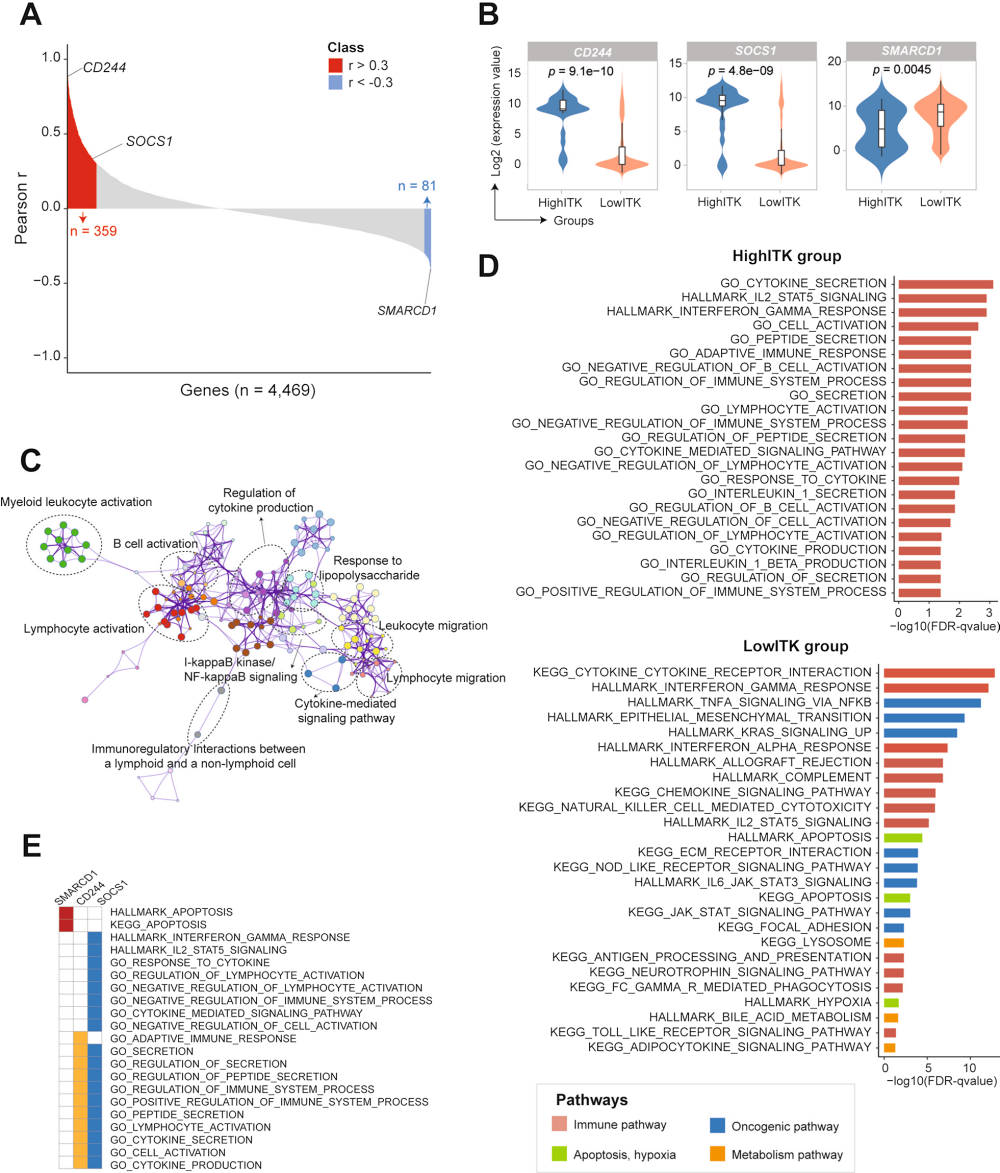
Gene Set Enrichment Analysis (GSEA) to identify key function of ITK. **A** The correlativity analysis between ITK expression and the TCGA gene data set (Pearso’s correlation test, r > 0.3 or r < 0.3 as threshold value), **B** The positive correlation genes (CD244 p = 9.1e−10, SOCS1 p = 4.8e−09) and negative correlation (SMARCD1 p = 0.0045) (t test). **C** The pathways analysis of the high correlated genes. **D**, **E** the cumulative distribution function analysis with high ITK level and low ITK level respectively

Since *ITK* expression affects the prognosis of ovarian cancer patients with regional and distant metastasis, we explored the correlation between *ITK* and 4,469 differentially expressed genes, and we discovered that 359 out of 4469 genes were positively correlated with *ITK* (Pearson r > 0.3) but 81 genes were negatively correlated with *ITK* (Pearson r < − 0.3; Fig. 5A and Additional file 4: Table S3). Moreover, alterations correlated with distant metastasis emereged with significantly increased expression in *SAMRCD1* in low-ITK group, but *CD244* and *SOCS1* in high-ITK group (*p* < 0.001; Fig. 5B and Additional file 5: Table S4), which indicated as oncogene or tumor suppressors in numerous cancers and potential therapeutic targets. Integrated analysis revealed dysregulated molecular processes including predominantly oncogenic signaling pathways in low-ITK group but immune related pathways in high*-*ITK group, which suggested *ITK* might inhibit distant metastasis in ovarian cancer (Fig. 5C, D). Furtherly, we identified the function and signaling pathways of the three key genes (*CD244, SOCS1* and *SAMRCD1*). As the favorable biomarkers, *CD244* and *SOCS1*, were mainly expressed in the immune pathway. Oppositely, the oncogene, *SMARCD1* was mainly expressed in apoptosis pathways (Fig. 5E). These results indicated that patients with poor prognosis predominantly had more oncogenic signaling pathways correlated with metastasis but immune-enriched pathways in low-ITK group.

### Deconvolution of the celluar compositon of ovarian cancer specimens revealed strong correlation between *ITK* and cytotoxic lymphocytes

**Fig. 6:**
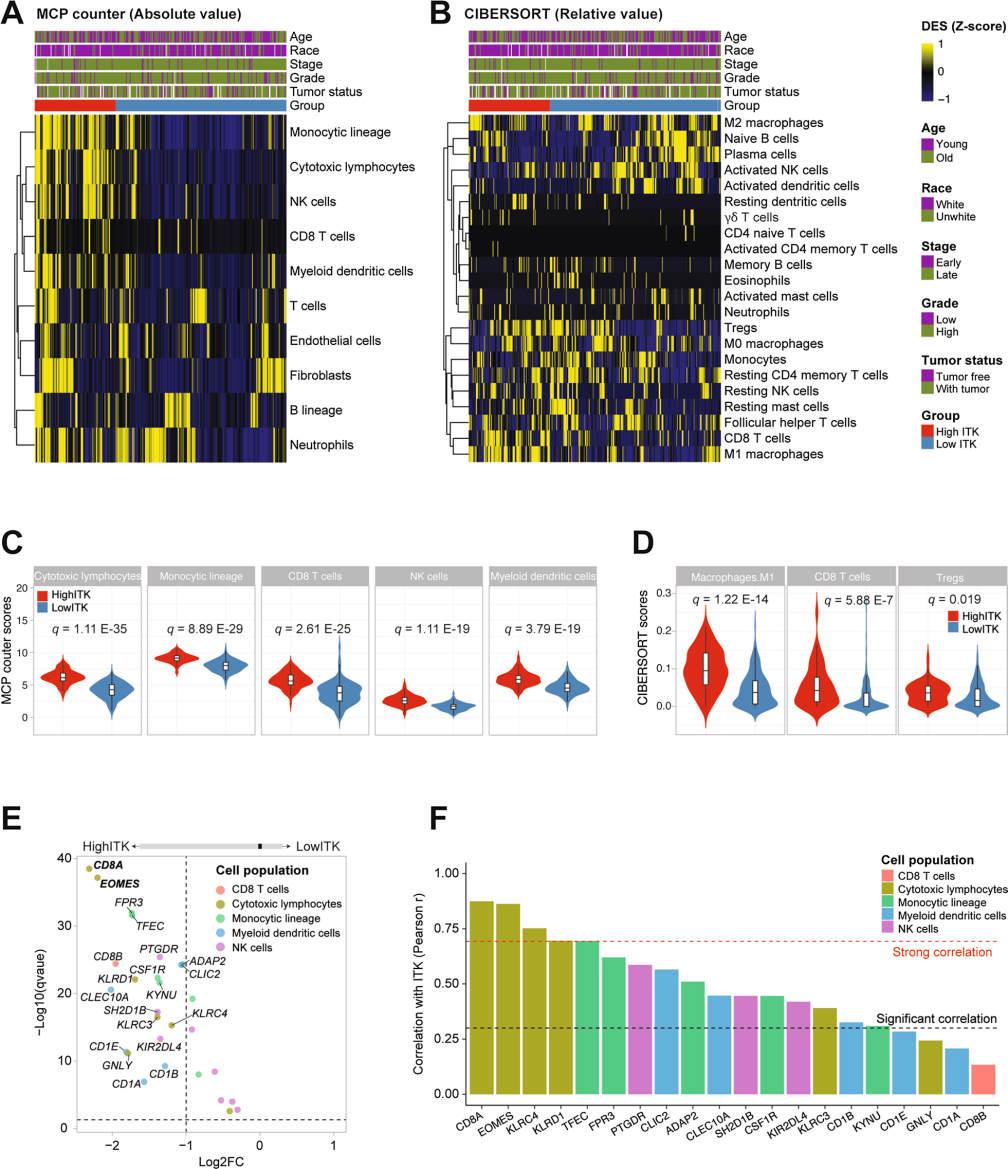
Specific immune landscape. **A**, **B** The yellow-black heatmaps showing quantification of ITK expression level and immune cells computed by MCP-counter and CIBERSORT-counter method. **C** The immune cells in high MCP counter scores (cytotoxic lymphocytes q = 1.11E−3.5, monocytic lineage q = 8.89E−29, CD8 T cells q = 2.61E−25, NK cells q = 1.11E−19 and myeloid dendritic cells q = 3.79E−19), **D** The immune cells in high CIBERSORT counter scores (M1 q = 1.22E−14, CD8 T cells q = 5.88E−7 and tregs q = 0.019), **E**, **F** The correlativity analysis between ITK expression and the marker of different immune cells (Pearso’s correlation test)

To identify the immune-related role of *ITK* in the tumor microenvironment (TME), we applied MCP-counter and CIBERSORT methods to estimate absolute abundance scores of 8 major immune cell types, endothelial cells and fibroblasts (Fig. 6A) and the relative cellular fraction of 22 immune cell types (Fig. 6B). The abundance scores from MCP-counter showed significantly lower abundance of cytotoxic lymphoctyes (*q* = 1.11E−3.5), monocytic lineage (*q* = 8.89E−29), CD8 T cells (*q* = 2.61E−25), NK cells (*q* = 1.11E−19) and myeloid dendritic cells (*q* = 3.79E−19) in low-ITK group compared with high-ITK group (Fig. 6C). And based on CIBERSORT method, macrophages.M1(*q* = 1.22E−14), CD8 T cells (*q* = 5.88E−7) and tregs (*q* = 0.019) were also significantly lower in low-ITK group (Fig. 6D). Furtherly, we analyzed all the markers in these two alogrithms corresponding to different immune cells between low and high-ITK groups, and we found that *CD8A*, *EOMES*, *KLRC4* and *KLRD1*, expressed in cytotoxic lymphocytes were strong correlation with *ITK* in the high-ITK group (Fig. 6E, F). These analysis suggested that ITK, as a favorable factor of TME, played an important role in the immune function of cytoxic lymphocytes.

## Discussion

Ovarian cancer has been extensively studied so far [33], yet the role of ITK in ovarian cancer has not been elucidated in many publications. However, an increasing number of ovarian cancer patients with metastasis from the public data sets had a poor prognosis[34, 35]. Therefore, we provided a landscape of *ITK* in ovarian cancer from the mouse model to patients, which could help us understand the correlation between *ITK* expression and the prognosis of patients with ovarian cancer.

The mouse model of ovarian cancer metastasis was successfully constructed by the CRISPR genome wide knock-out library, and the genes were sequenced through RIGER method. Our data showed that *ITK* expression was significantly inhibited in the metastatic mouse model, which suggested its essential role in the occurrence and development of ovarian cancer metastasis. Then we explored the correlation between low *ITK* expression and its DNA mutation. We observed two truncating mutations (X238 and X505) and four shallow deletions (D202A, L254V, A261P, and X505) involved in the mutated *ITK*. The multi-locus and multi-modal mutations may lead to being a low expression of *ITK* in ovarian cancer [36]. Next, we investigated whether the alteration of *ITK* expression in the ovarian cancer metastasis mouse model was consistent with that in the ovarian cancer patients. We analyzed and validated these results in TCGA as well as GSE38666 data set. The expression of *ITK* was significantly lower in patients with ovarian cancer than normal samples, especially in the patients with un-regional metastasis. Moreover, these patients with metastasis had poor prognosis in the low-ITK group than those in high-ITK group. All the data suggested that *ITK* was a tumor suppressor gene, especially a gene that inhibited cancer metastasis both in mouse models and patients. There was a strong correlation between the *ITK* expression and the prognosis of patients. With the increase of gene expression value, the overall survival rate of patients is gradually increased. Then the multivariable Cox regression model was used to evaluate the independency of *ITK* in predicting prognosis in TCGA and GEO data sets. The similar results in both data sets showed that the HRs of patients with low *ITK* expression was more significant than those with high *ITK* expression, which suggested that the expression of *ITK* can be used as an independent prognostic factor to predict the prognosis of patients with ovarian cancer. Furtherly, we evaluated the diagnostic power of *ITK* expression by using ROC curve analysis. And we found that *ITK* could add the diagnostic power of the clinical factors, although the AUC value of *ITK* was not very high in each data set.

To more fully assess the function of ITK in ovarian cancer, we furtherly performed correlation analysis and found that the *ITK* expression was positively correlated with 359 genes (including cancer suppressor gene *CD244* and *SOCS1, r* > *0.3*) and negatively associated with 81 genes (including oncogene *SMARCD1, r* < − *0.3)*. In a previous study, the receptor encoded by the *CD244* gene was thought to modulate NK-cell cytolytic activity and predominantly displayed inhibitory signaling in tumor-associated immune cells. The receptors' activity and expression and their ligands were correlated with tumor progression, prognosis, and inflammatory responses [37]. Also, it has been reported that the expression level of *SOCS1* was significantly increased in human ovarian cancer and might function as a diagnostic biomarker [38]. Our results showed that *ITK* expression was positively correlated with cancer suppressor gene *CD244* (p = 9.1e−10) and *SOCS1* (p = 4.8e−09) among patients with a favorable prognosis, suggesting that the function of *ITK* may be similar with *CD244* and *SOCS1*. Moreover, oncogene *SMARCD1* was upregulated in ovarian cells exposed to ascites, allowing a non-stimulatory effect on cancer cell migration [39]. The *ITK* expression was negatively correlated with *SMARCD1(p* = *0.0045)* in patients with ovarian metastases. All these data showed that *ITK* could act as a cancer suppressor gene in patients with ovarian cancer and had a similar function to *CD244* and *SOCS1*.

We integrated the pathways enrichment analysis between *ITK* and another related genes. The results revealed that these genes in high-ITK group were positively correlated with the immune pathway, and genes in low-ITK group were associated with apoptosis and oncogenic pathway. This indicated that patients with low *ITK* expression were with poor prognosis, mainly because of the factors involved in the cancer development. In contrast, patients with high *ITK* expression were with favorable prognosis, mainly due to have more factors involved in immune pathways. Finally, we continued to verify the *ITK* expression in immunity. The results showed that high *ITK* expression was mainly enriched in different immune cells, such as cytotoxic lymphocytes, monocytic lineage, NK cells, and myeloid dendritic cells. Moreover, *ITK* expression was positively correlated with markers of these immune cells in patients with favorable prognosis. Generally, it suggested that *ITK* was indeed involved in the immune function and produced sound prognostic effects.

The biological validation is in some way weak in this study. We understand that it is better to reveal the potential *ITK* transcriptional mechanism by detecting the RNA and protein expression in corresponding cancer cells and tumor tissues. However, we predominantly focused on exploring the clinical analysis with bioinformatics methods to reveal the mechanism of ITK with low expression in the ovarian cancer patients. And the further efforts will be paid to validate the discoveries about the expression and function of ITK with biological experiments in the next study.

In summary, we identified a strong correlation between differential *ITK* expression and ovarian cancer development and prognosis, which was verified by TCGA and GEO data sets analysis. Moreover, our enrichment analysis results highlighted the prognostic role of *ITK* in ovarian cancer were involved in immunology function. Novel prognostic markers are urgently required. This research will provide a framework for future study and a factor for the diagnosis of ovarian cancer, which allows us to define the prognosis status of ovarian patients.

## Supplementary Information

**Additional file 1: Table S1.** Basical characteristics.

**Additional file 2: Figure S1.** A), The numbers of metastatic nodules on the surfaces of intestines in CRISPR-Vec and CRISPR-Lib in the third round screening (White arrow represents tumor in primary site; blue arrow represents metastatic nodules), B) Bioluminescence.

**Additional file 3: Table S2.** All genes ttest result between Regional and Unregional groups.

**Additional file 4: Table S3.** Correlation analysis among the 4751 genes.

**Additional file 5: Table S4.** Key genes correlated with ITK.

## Data Availability

All data generated or analyzed during this are included in this article.
