# Supplementary material for: Cytotoxic lymphocytes-related gene ITK from a systematic CRISPR screen could predict prognosis of ovarian cancer patients with distant metastasis
Source: J Transl Med. 2021 Oct 26;19:447. doi: 10.1186/s12967-021-03119-3 (PMC8549276; doi:10.1186/s12967-021-03119-3)
Supplement: Supplementary file 2 — Additional file 2: Figure S1. A), The numbers of metastatic nodules on the surfaces of intestines in CRISPR-Vec and CRISPR-Lib in the third round screening (White arrow represents tumor in primary site; blue arrow represents metastatic nodules), B) Bioluminescence. [file 12967_2021_3119_MOESM2_ESM.docx]

**Additional file**

**Cytotoxic lymphocytes-related gene ITK from a systematic CRISPR screen could predict prognosis of ovarian cancer patients with distant metastasis**

Mengyao Xu^1,#^, Shan Huang^1,#^, Jiahui Chen ^3^, Wanxue Xu^1^, Rong Xiang^1^ , Yongjun Piao^1,2,*^, Shuangtao Zhao^4,*^

^1^School of Medicine, Nankai University, Tianjin 300071, China.

^2^Tianjin Key Laboratory of Human Development and Reproductive Regulation, Nankai University Affiliated Hospital of Obstetrics and Gynecology, Tianjin, China

^3^Department of Nuclear Medicine and PET/CT-MRI Center, The First Affiliated Hospital of Jinan University, 613 West Huangpu Road, Tianhe District, Guangzhou 510630, China.

^4^No.2 Department of Thoracic Surgery, Beijing Tuberculosis and Thoracic Tumor Research Institute/Beijing Chest Hospital, Capital Medical University, Beijing 101149, China

^#^ Equal contribution

*Co-corresponding author:

1) Shuangtao Zhao, Tel.: +86-13520790625, Email: zst-1981@163.com

2) Yongjun Piao, Tel.: +86-18526062102, Email: ypiao@nankai.edu.cn

Figure S1 A), The numbers of metastatic nodules on the surfaces of intestines in CRISPR-Vec and CRISPR-Lib in the third round screening (White arrow represents tumor in primary site; blue arrow represents metastatic nodules), B) Bioluminescence in primary site and metastatic organ in CRISPR-Vec and CRISPR-Lib.
